# Janus Kinase Inhibition Ameliorates Ischemic Stroke Injury and Neuroinflammation Through Reducing NLRP3 Inflammasome Activation *via* JAK2/STAT3 Pathway Inhibition

**DOI:** 10.3389/fimmu.2021.714943

**Published:** 2021-07-22

**Authors:** Hua Zhu, Zhihong Jian, Yi Zhong, Yingze Ye, Yonggang Zhang, Xinyao Hu, Bei Pu, Lijuan Gu, Xiaoxing Xiong

**Affiliations:** ^1^Department of Neurosurgery, Renmin Hospital of Wuhan University, Wuhan, China; ^2^Central Laboratory, Renmin Hospital of Wuhan University, Wuhan, China; ^3^Department of Oncology, Renmin Hospital of Wuhan University, Wuhan, China; ^4^Department of Anesthesiology, Renmin Hospital of Wuhan University, Wuhan, China

**Keywords:** ruxolitinib, ischemic stroke, neuroinflammation, NLRP3 inflammasome, JAK2/STAT3

## Abstract

**Background:**

Inflammatory responses play a multiphase role in the pathogenesis of cerebral ischemic stroke (IS). Ruxolitinib (Rux), a selective oral JAK 1/2 inhibitor, reduces inflammatory responses *via* the JAK2/STAT3 pathway. Based on its anti-inflammatory and immunosuppressive effects, we hypothesized that it may have a protective effect against stroke. The aim of this study was to investigate whether inhibition of JAK2 has a neuroprotective effect on ischemic stroke and to explore the potential molecular mechanisms.

**Methods:**

Rux, MCC950 or vehicle was applied to middle cerebral artery occlusion (MCAO) mice *in vivo* and an oxygen-glucose deprivation/reoxygenation (OGD/R) model *in vitro*. After 3 days of reperfusion, neurological deficit scores, infarct volume and brain water content were assessed. Immunofluorescence staining and western blots were used to measure the expression of NLRP3 inflammasome components. The infiltrating cells were investigated by flow cytometry. Proinflammatory cytokines were assessed by RT-qPCR. The expression of the JAK2/STAT3 pathway was measured by western blots. Local STAT3 deficiency in brain tissue was established with a lentiviral vector carrying STAT3 shRNA, and chromatin immunoprecipitation (ChIP) assays were used to investigate the interplay between NLRP3 and STAT3 signaling.

**Results:**

Rux treatment improved neurological scores, decreased the infarct size and ameliorated cerebral edema 3 days after stroke. In addition, immunofluorescence staining and western blots showed that Rux application inhibited the expression of proteins related to the NLRP3 inflammasome and phosphorylated STAT3 (P-STAT3) in neurons and microglia/macrophages. Furthermore, Rux administration inhibited the expression of proinflammatory cytokines, including TNF-α, IFN-γ, HMGB1, IL-1β, IL-2, and IL-6, suggesting that Rux may alleviate IS injury by inhibiting proinflammatory reactions *via* JAK2/STAT3 signaling pathway regulation. Infiltrating macrophages, B, T, cells were also reduced by Rux. Local STAT3 deficiency in brain tissue decreased histone H3 and H4 acetylation on the NLRP3 promoter and NLRP3 inflammasome component expression, indicating that the NLRP3 inflammasome may be directly regulated by STAT3 signaling. Rux application suppressed lipopolysaccharide (LPS)-induced NLRP3 inflammasome secretion and JAK2/STAT3 pathway activation in the OGD/R model *in vitro*.

**Conclusion:**

JAK2 inhibition by Rux in MCAO mice decreased STAT3 phosphorylation, thus inhibiting the expression of downstream proinflammatory cytokines and the acetylation of histones H3 and H4 on the NLRP3 promoter, resulting in the downregulation of NLRP3 inflammasome expression.

## Introduction

Stroke is known to be one of the leading causes of disability and death (1). The most common type of stroke is ischemic stroke (IS); however, the pathogenesis of IS is not fully clear, and treatment is limited. It is well known that neurons and microglia/macrophages play important roles in the production of proinflammatory cytokines and in the inflammatory cascades triggered by IS (2). Previous evidence suggests that cerebral ischemia leads to cell death, including neurons, which secrete inflammatory cytokines and reactive oxygen species, eventually leading to the activation and infiltration of microglia/macrophages (3). In addition, microglia/macrophages can be activated directly by neuronal death. Inflammatory cytokines simultaneously promote the entry of leukocytes into brain tissue, generating an inflammatory cascade response that causes activated microglia and infiltrating leukocytes to produce more inflammatory mediators, such as the nucleotide-binding oligomerization domain-like receptor protein 3 (NLPR3) inflammasome, which cause brain edema and hemorrhage, increase blood-brain barrier damage, and promote more neuronal death (3). Although various anti-inflammatory agents have shown great potential in alleviating ischemic injury in animal models of IS, most have subsequently failed in clinical trials (4). Hence, new therapies aimed at rescuing neuronal cells and inhibiting inflammation are urgently needed to improve therapeutic outcomes.

Recently, in COVID-19 (a disease that induced an inflammatory cascade) patients with serious systemic hyperinflammation, ruxolitinib (Rux) was reported to exert therapeutic effects by interfering with numerous proinflammatory cytokines *via* inhibition of the Janus kinase/signal transducer and activator of transcription (JAK/STAT) pathway to intervene in the detrimental cytokine release syndrome regulated by pulmonary inflammation (5). Rux, an orally bioavailable, selective inhibitor of JAK 1/2, is used to treat polycythemia vera (6), recalcitrant dermatomyositis (7), and myelofibrosis (8), among other diseases. Rux also has antitumor (9), immunosuppressive and anti-inflammatory functions (10). The key to the broad anti-inflammatory activity of Rux is its ability to inhibit numerous proinflammatory cytokines, such as IFN-γ, TNF-α, IL-1, IL-6, IL-8, IL-12 and GM-CSF, associated with the innate cytokine storm (11). Hence, we hypothesize that Rux may inhibit the inflammatory cascade in IS. In addition, the NLRP3 inflammasome can trigger the cytokine storm in the COVID-19 inflammatory cascade (12, 13). Moreover, the NLRP3 inflammasome drives inflammatory reactions after IS (14). Our previous study demonstrated that inhibition of the NLRP3 inflammasome by meisoindigo had a neuroprotective effect during IS (15). The inflammatory reaction can be alleviated *via* suppression of the NLRP3 inflammasome and the NOX4/JAK2/STAT3 pathway (16). However, whether Rux can inhibit the NLRP3 inflammasome in IS remains unknown.

JAK2, a member of the protein-tyrosine kinase family, is an important regulator of many physiological and pathological processes, including the inflammatory response and cell proliferation and differentiation. Other signaling molecules, such as STAT1, STAT3, and STAT5, are also regulated by JAK2 (17). Specifically, the phosphorylation of STAT3 can be induced by JAK2 (18). There is mounting evidence that the JAK2/STAT3 pathway exerts an important effect on the inflammatory reaction (19, 20). Changes in the JAK2/STAT3 pathway affect the expression of many cytokines, such as TNF-α and IL-6 (20, 21). Moreover, the JAK2/STAT3 pathway contributes to brain damage caused by ischemia/reperfusion (22). It has been reported that overactivation of STAT3 located at microglia exaggerates microglial activation and neuroinflammation after IS (23). Moreover, JAK2/STAT3 inhibition can ameliorate cerebral ischemic injury and neuroinflammation by inhibiting microglial mitochondrial (24, 25). However, whether inhibition of the JAK2/STAT3 pathway inhibits proinflammatory cytokines that are detrimental in IS and the associated mechanisms are still unclear. Additionally, the impact of Rux on NLRP3 inflammasome expression and the JAK2/STAT3 pathway, as well as the direct interaction between the NLRP3 inflammasome and the JAK2/STAT3 pathway remain to be elucidated.

In this study, we explored whether the administration of Rux exerts a neuroprotective effect in middle cerebral artery occlusion (MCAO) mice *in vivo* and in an oxygen-glucose deprivation/reperfusion (OGD/R) model *in vitro*. Then, we investigated whether Rux interferes with the expression of NLRP3 inflammasome components after IS and whether the JAK2/STAT3 pathway is involved in the neuroprotective and anti-inflammatory effects of Rux on IS. Finally, the interplay between the NLRP3 inflammasome and the JAK2/STAT3 pathway following IS was studied.

## Methods

### Animals

The animal protocols were approved by the Animal Care and Use Committee Guidelines of Wuhan University. To eliminate the influence of gender differences on IS, male C57BL/6J mice (25-30 g) were purchased from Wuhan University Center for Animal Experiments and group-housed at 25 ± 1°C and 65 ± 5% humidity on a 12/12-h light/dark cycle with free access to water and food.

### Drug Administration

Rux (C10891361, Shanghai Chemical Industry Park, Shanghai, China) was dissolved in 30% polyethylene glycol 300 (PEG300) and 3% dimethyl sulfoxide (DMSO) as previously described (26) and diluted with distilled water (dH2O) to the required concentrations. In the first experiment, mice were treated 30 minutes after MCAO with 4 different doses of Rux (30, 60, 90, and 120 mg/kg) by oral gavage twice daily for three days. Based on the results of the first experiment, mice were treated with 90 mg/kg Rux in subsequent experiments, which is consistent with the treatment regimen and dose of Rux used in previous studies on other disease models (26, 27). To investigate the therapeutic effects of prophylactic administration and therapeutic administration, 90 mg/kg of Rux was also administrated by oral gavage twice daily for three days before surgery. MCC950 (PZ0280, Sigma-Aldrich, St. Louis, MO, USA), an inhibitor of the NLRP3 inflammasome, was dissolved in sterile saline and administered 30 minutes after MCAO (50 mg/kg, i.p.) for 3 days as a positive control (28). The control group was administered vehicle solvents containing no Rux or MCC950.

### Lentivirus Administration

A lentiviral vector carrying STAT3 shRNA (Lenti-STAT3) was constructed by GeneChem (Shanghai, China) as previously described (29). The siRNA oligonucleotide was 5’CcggCCTGAGTTGAATTATCAGCTTCTCGAGAAGCTGATAATTCAACTCAGGTTTTTg3’, targeting mouse STAT3 (NM_213659). Lenti-STAT3 was injected into the mouse brain as previously reported (30, 31). In brief, 4 injections were given at the following coordinates: 0.5 mm anterior to bregma, 2.0- or 3.0-mm lateral (right) to the sagittal suture, and 1.0 or 2.8 mm from the surface of the skull. Concentrated lentivirus (0.5 μL in total, 2*10^9^ transducing units/mL) was injected at a speed of 0.5 μL/min. Lenti-GFP served as the control. After the lentivirus injection, the needle was kept in position for five minutes and then removed slowly so that the lentivirus was absorbed well. Sham and IS surgeries were carried out 4 days after lentivirus microinjection.

### IS Model

The IS model was established under isoflurane anesthesia as previously described (32, 33). During this procedure, the rectal temperature of each mouse was stabilized at 36.5 ± 0.5°C with a thermostatic heating pad. Unilateral MCAO was induced on the left side through occlusion of the origin of the left middle cerebral artery with a 6.0-mm silk (Doccol, Corp., Redlands, CA, USA). One hour after occlusion, reperfusion was initiated by removing the monofilament. Sham control mice underwent the same surgical operation but without ligation.

### Infarct Volume Measurement and Neurological Evaluation

As previously described, neurological deficits were assessed 3 days after MCAO according to a neurologic grading scale: 0, no neurological deficit; 1, flexion of contralateral forelimb; 2, severe forelimb flexion and decreased resistance to lateral push without circling; 3, unidirectional circling; and 4, without spontaneous motor activity (32). After euthanasia with an overdose of isoflurane, the animals were decapitated. Then, their brains were collected and cut into 2 mm coronal sections. The sections were immediately incubated in 2% 2,3,5-triphenyltetrazolium chloride (TTC) for staining. The infarct size (percentage of hemisphere size) was evaluated and corrected for edema by an evaluator in a blinded manner by using ImageJ (version 1.61, NIH, Bethesda, MD, USA), as described in our previous works (33, 34).

### Brain Water Content Measurement

Three days after surgery, ischemic brain edema was measured based on the formula: (wet weight-dry weight)/wet weight × 100% (35). Briefly, brain tissues were collected without cardiac perfusion, the wet weights were immediately determined using an analytical balance, and then the dry weights were assessed after drying for 24 h at 105°C.

### Immunofluorescence Staining

Animals were euthanized 3 days after MCAO and perfused with icy PBS, followed by 4% paraformaldehyde, as previously described (33). The brains were taken and postfixed in 4% paraformaldehyde for 48 hr and cut into 50-µm coronal slices. PBS (0.1 M, pH 7.4) was used for all incubations and washes. Immunofluorescence staining was performed under moderate shaking. The slices were blocked with blocking buffer (0.1 M PBS, 5% fetal bovine serum (FBS), and 0.3% Triton X) for 1 hour. Then, the sections were incubated with the following antibodies at 4°C overnight: anti-P-STAT3 (diluted 1:200; ab76315, Abcam, Cambridge, UK), anti-NLRP3 (diluted 1:200; ab4207, Cell Signaling Technology (CST), Boston, MA, USA), anti-CD68 (diluted 1:200; MCA1957, AbD Serotec, Oxford, UK), anti-GFAP (diluted 1:200; CST, Boston, MA, USA), anti-MPO (diluted 1:200; Servicebio, Wuhan, China) and anti-NeuN (diluted 1:200; ab104224, Abcam, Cambridge, UK). Then, the sections were thoroughly washed and incubated at room temperature for two hours with an Alexa Fluor 488-conjugated antibody (diluted 1:200 for P-STAT3, NeuN, GFAP, and CD68; Millipore, MA, USA) or Alexa Fluor 594-conjugated antibody (diluted 1:200 for NLRP3 and MPO; Millipore, MA, USA). After washing, the nuclei were stained with DAPI. The sections were observed under an epifluorescence microscope (Olympus Optical, Japan) covering a total area of 0.19 mm^2^. Five different fields (0.50 to 0.38 mm in the penumbra of the brain tissues) per mouse and five mice per group were assessed. Immunoreactive cell counts in predetermined regions were determined by evaluators in a blinded manner using ImageJ.

### Real-Time Quantitative PCR

Total RNA was extracted from the peri-infarct area of the ischemic hemisphere with TRIzol (#9109, TaKaRa, Shiga, Japan), as previously described (15). The PrimeScript RT Reagent Kit (TaKaRa, Japan) was used for reverse transcription based on the manufacturer’s protocol. The primers (Beijing Genomics Institute) used to amplify the cDNA are listed in **Table 1**. The cDNA was mixed with SYBR Premix Ex Taq2 (TaKaRa, Japan) and synthetic primers for real-time quantitative PCR. The PCR conditions were selected based on the manufacturer’s protocol and were as follows: 2 min at 50°C; 10 min at 95°C; and 45 cycles of 10 s at 95°C, 10 s at 60°C and 15 s at 72°C. Relative mRNA expression levels were quantified by normalization to the expression of the internal reference GAPDH. Gene expression levels are shown as fold changes compared to the sham group.

**Table 1:** Primers for RT-PCR.

Genes		Primers (5’ –3’)
**INF-γ**	F	CACGCCGCGTCTTGGT
	R	TCTAGGCTTTCAATGAGTGTGCC
**IL-1β**	F	TCTAGGCTTTCAATGAGTGTGCC
	R	ATCTTTTGGGGTCCGTCAACT
**TNF-α**	F	GACGTGGAACTGGCAGAAGAG
	R	TTGGTGGTTTGTGAGTGTGAG
**IL-10**	F	GACCAGCTGGACAACATACTGCTAA
	R	GATAAGGCTTGGCAACCCAAGTAA
**IL-6**	F	GGTCCAGTTGCCTTCTCCC
	R	GCAACAAGGAACACCACGG
**IL-4**	F	GCAACAAGGAACACCACGG
	R	AAGCACGGAGGTACATCACGT
**IL-2**	F	ACCCTTGCACTCATGGCAAA
	R	TCAATTCTGTAGCCTGCTTGGG
**HMGB1**	F	TTTCAAACAAAGATGCCACA
	R	GTTCCCTAAACTCCTAAGCAGATA
**GAPDH**	F	AGGTCGGTGTGAACGGATTTG
	R	TGTAGACCATGTAGTTGAGGTCA

### ChIP Assays

ChIP assays were performed as per a previously reported method (36). Briefly, three days after MCAO, the brains of lentivirus-injected mice were removed and placed in 1% formaldehyde for 15 min. The tissue was homogenized and placed in lysis buffer. Then, the DNA was sheared by sonication. After adding ChIP dilution buffer to the DNA sample, 5 μl of sample was saved as input. Anti-P-STAT3 (Abcam, UK), anti-acetylated histone H3 (ac-H3) (Abcam, UK) or anti-acetylated histone H4 (ac-H4) (Abcam, UK) antibodies were added to precleared chromatin solution overnight at 4°C. “IgG” immunoprecipitation served as a negative control. On the second day, the DNA was purified from the complexes, and input fractions following the antibody/DNA complexes were captured, washed, eluted, and reverse cross-linked. The precipitated DNA was subjected to quantitative real-time PCR, and the amplified fragments obtained from the sample incubated with anti-P-STAT3 were then analyzed by 1.5% (w/v) agarose gel analysis. The ChIP/input ratio was measured. Primers 5′-GGGACCAAATTGAGG GCTTC-3′ and 5′-TCAACGTCACCAGTCCTCAGA-3′ were designed to amplify a −2935/−2828 region relative to the mouse NLRP3 promoter transcription start site containing the STAT3-binding site.

### OGD/R *In Vitro*

Before initiating OGD/R injury, mouse hippocampal cells (HT-22 cells, Center for Type Culture Collection, Hubei, China) were cultured in an incubator with 5% CO_2_ (37°C) in DMEM supplemented with 1% antibiotic solution (GNM15140, Genome, China) and 10% FBS. Afterward, HT-22 cells were incubated in a hypoxic incubator (94% N_2_, 5% CO_2_ and 1% O_2_, 37°C) for the optimal time to induce OGD injury. Then, the cells were allowed to recover under normoxic conditions (OGD restoration) for 12 h. Cells maintained in DMEM without oxygen deprivation served as the control group.

### *In Vitro* Drug Administration and Cell Viability Measurement

The viability of HT22 cells was assessed by the Cell Counting Kit (CCK)-8 cytotoxicity assay (Dojindo Laboratory, Japan). Briefly, HT-22 cells were seeded in a 96-well plate in DMEM containing 10% FBS. HT-22 cells were treated with lipopolysaccharide (LPS) (1 µg/mL) as described in our previous study (15) and different concentrations of Rux (1, 2, 3, 4, 5, or 6 µM) prior to the induction of OGD. After the medium was removed, CCK-8 reagent (10 µl) was applied to each well and incubated for 2 h at 37°C. An automatic microplate reader (Albert, VT, USA) was used to determine the absorbance at 450 nm.

### Western Blotting

Three days after reperfusion, total protein was harvested from HT-22 cells subjected to different treatments and ipsilateral brain tissues, as previously described (32). The tissues were homogenized and lysed on ice in ice-cold RIPA buffer (Applygen, Beijing, China) supplemented with proteinase and phosphatase inhibitors (Servicebio, Wuhan, China). Then, the protein samples were separated on gels by electrophoresis and transferred to PVDF membranes. The membranes were incubated in blocking buffer for one hour and then incubated with a mouse anti-acetylated histone H4 (ac-H4) (diluted 1:1000; Abcam, Cambridge, UK), anti-AQP4 (diluted 1:1000; 2042744, Millipore, Billerica, MA, USA), anti-NLPR3 (diluted 1:1000; ab4207, Abcam, Cambridge, UK), anti-JAK2 (diluted 1:1000; ab108596, Abcam, Cambridge, UK), anti-ASC (diluted 1:1000; 67824, CST, Boston, MA, USA), anti-CL-caspase-1 (diluted 1:500; 89332, CST, Boston, MA, USA), anti-STAT3 (diluted 1:1000; ab8378, Abcam, Cambridge, UK), anti-P-STAT3 (diluted 1:1000; ab32536, Abcam, Cambridge, UK), anti-acetylated histone H3 (ac-H3) (diluted 1:500; Abcam, Cambridge, UK), anti-phosphorylated JAK2 (diluted 1:1000; ab195055, Abcam, Cambridge, UK), anti-IL-1β (diluted 1:500; ab8320, Abcam, Cambridge, UK), anti-IL-18 (diluted 1:500; ab207324, Abcam, Cambridge, UK) or anti-β-Tubulin (diluted 1:1000; 2148S, CST, Boston, MA, USA) antibody at 4°C overnight. Then, the membranes were incubated with an IRDye-labeled secondary antibody (diluted 1:10000; Li-Cor Bioscience, USA) for 1 hour. Images were assessed with Odyssey software (LI-COR, Lincoln, NE, USA). Afterward, the optical intensity of each band was analyzed and normalized to the optical density of the β-tubulin band using ImageJ.

### Flow Cytometry

After 3 days of perfusion, mice were euthanized with excess isoflurane and transcardially perfused with 50 mL cold PBS. Ipsilateral ischemic hemispheres were collected and homogenized, on ice, with FACS buffer (PBS containing 1% fetal bovine serum) at a volume of 7 mL, and then mixed with 3 mL 90% Percoll (GE Healthcare, Little Chalfont, UK) and loaded with 1 mL 70% Percoll. The cell suspension was then centrifuged at 500 ×g, for 30 min, at 4°C. The collected cells were washed with FACS buffer and then labeled with antibodies against CD3, CD45, CD11b, and B220 (Bio Legend, San Diego, CA), on ice, for 30 min, in the dark. About 100,000 cells were collected for analysis on a CytoFLEX flow cytometer (Beckman Coulter). Next, the expression of cell surface molecules was measured and analyzed using CytExpert software (v2.3, Beckman Coulter) and FlowJo software (v10.0.7, Tree Star, Ashland, OR).

### Statistical Analysis

The data are presented as the means ± SD and were analyzed by one-way ANOVA and then subjected to Tukey’s test for multiple comparisons. *P* < 0.05 was regarded as statistically significant.

## Results

### Rux Decreases the Infarct Size, Neurological Scores, and Brain Edema 3 Days After IS

**Figure 1:**
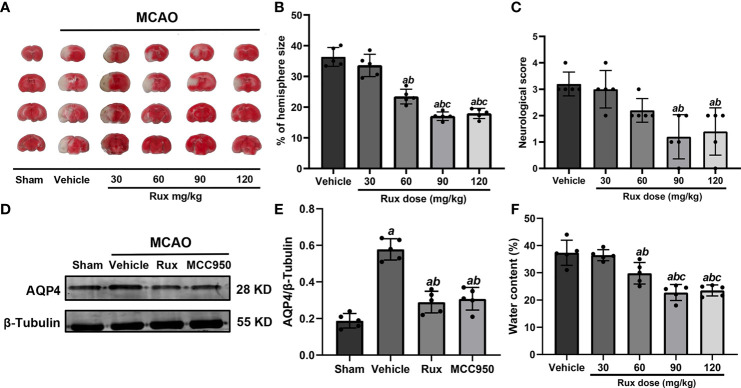
Rux reduced infarct size, neurological deficits and cerebral edema in mice after MCAO. **(A)** Representative TTC-stained slices showing infarction in vehicle- and Rux-treated mice. **(B)** Quantitative analysis of the infarct size is presented as a percentage of the contralateral hemisphere size 3 days after stroke. **(C)** Neurological scores were determined 3 days after MCAO. **(D)** Representative western blots showing that Rux application reduced AQP4 expression. **(E)** Quantification of AQP4 expression. **(F)** Quantification of water content. Means ± SD. n = 5 in each group. ^a^*P* < 0.05 versus the vehicle group; ^b^*P* < 0.05 versus the 30 mg/kg Rux group; ^c^*P* < 0.05 versus the 60 mg/kg Rux group.

To determine whether Rux can protect against ischemic brain injury, we established an IS mouse model and treated the mice with Rux. Some mice from each group died within 3 days after MCAO (**Figure S1**). We subsequently measured the infarct volume and brain edema and assessed the neurologic scores of surviving animals 3 days after IS. The results showed that compared with vehicle, 30 mg/kg Rux did not affect the neurological deficits or infarct volume, as determined by TTC staining of coronal sections (**Figures 1A–C**). However, compared with vehicle administration (36.32 ± 3.07), administration of 60 (22.44 ± 2.40, *P* < 0.001), 90 (17.04 ± 1.38, *P* < 0.001) and 120 (17.94 ± 1.66, *P* < 0.001) mg/kg Rux significantly decreased the infarct size (% of hemisphere size) 3 days after MCAO (**Figures 1A, B**). Similarly, 90 (1.20 ± 0.84, *P* = 0.002) and 120 (1.40 ± 0.89, *P* = 0.004) mg/kg Rux ameliorated neurological deficits compared with the neurological score of the vehicle group (3.2 ± 0.45) (**Figure 1C**).

AQP4, an aquaporin protein, exerts a crucial effect on the pathogenesis of brain edema and affects the prognosis of IS. Hence, we examined whether Rux affects cerebral edema and the expression of AQP4 after MCAO. 60 (29.80 ± 3.96, *P* = 0.012), 90 (22.78 ± 2.92, *P* < 0.001) and 120 (23.52 ± 2.02, *P* < 0.001) mg/kg Rux significantly decreased brain edema (water content) and compared with vehicle (27.36 ± 4.62) (**Figure 1F**). Rux (0.290 ± 0.06, *P* < 0.001) also reduced AQP4 expression compared with vehicle (0.578 ± 0.06) (**Figures 1D, E**). Moreover, the results demonstrated that 90 mg/kg Rux had a better protective effect against focal cerebral ischemia-reperfusion injury (CIRI) (**Figures 1A–C, F**) than 30 and 60 mg/kg Rux. In addition, there was no significant difference in cerebral infarct size between prophylactic administration and therapeutic administration of 90 mg/kg Rux (17.48 ± 0.92 vs 17.04 ± 1.38, *P* = 0.449) (**Figure S2**). Thus, therapeutic administration of 90 mg/kg Rux was selected for subsequent experiments. MCC950, an NLRP3 inflammasome inhibitor, had effects similar to Rux. Therefore, we hypothesized that Rux may decrease the expression of NLRP3 inflammasome components after IS.

### Rux Treatment Inhibits the Expression of NLRP3 Inflammasome Components 3 Days After Stroke

**Figure 2:**
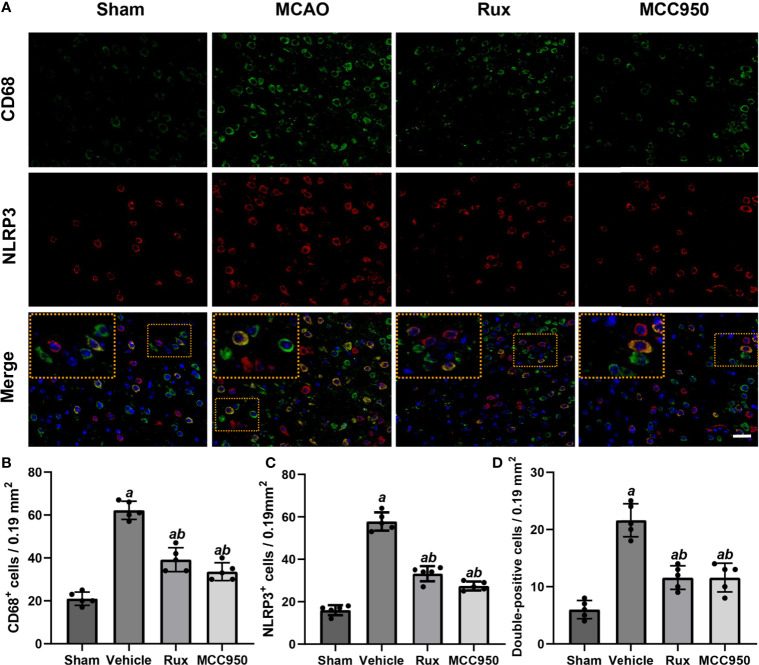
Rux treatment decreased the number of NLRP3^+^ and CD68^+^ cells in the ischemic penumbra. **(A)** Representative images of NLRP3 and CD68 immunostaining in the ischemic penumbra of sham-, vehicle-, Rux- and MCC950-treated mice 3 days after stroke. **(B, C)** Statistical analysis of NLRP3^+^ and CD68^+^ cells. **(D)** Quantification of double-positive cells in the peri-ischemic region. Means ± SD. n = 5. ^a^*P* < 0.01 versus sham; ^b^*P* < 0.01 versus vehicle. Scale bar: 50 µm.

**Figure 3:**
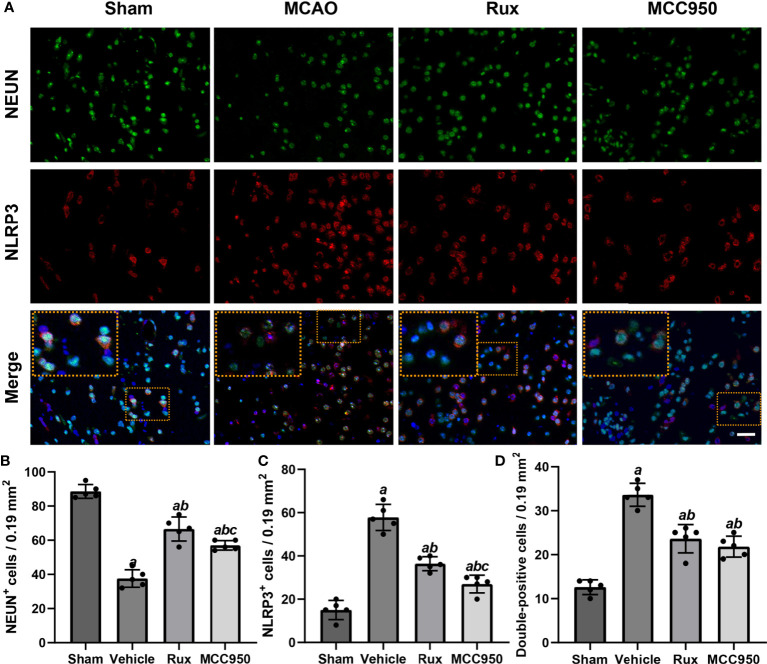
Rux treatment reduced the number of NLRP3^+^ cells and increased the number of NeuN^+^ cells in the ischemic penumbra 3 days after MCAO. **(A)** Representative immunostaining of NLRP3 and NeuN in the penumbra zone in sham-, vehicle-, Rux- and MCC950-treated mice. **(B, C)** Statistical analysis of NLRP3^+^ and CD68^+^ cells. **(D)** Quantification results of double-positive cells in the peri-ischemic region. Means ± SD. n = 5. ^a^*P* < 0.01 versus sham; ^b^*P* < 0.01 versus vehicle; ^c^*P* < 0.05 versus Rux. Scale bar: 50 µm.

**Figure 4:**
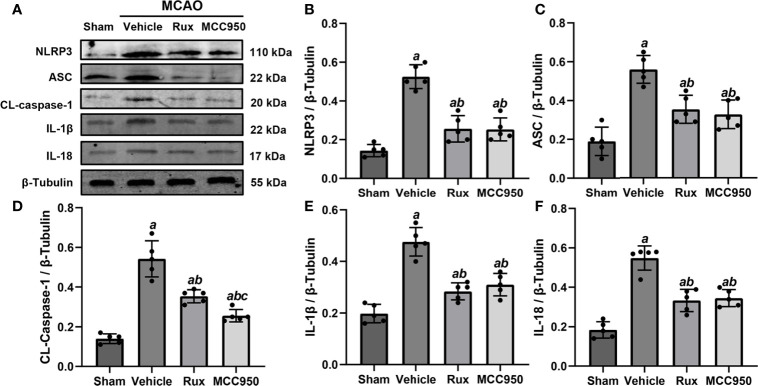
Rux treatment suppressed NLRP3 inflammasome-associated proteins. **(A)** Representative western blots showing that administration of Rux reduced the expression of NLRP3, ASC, CL-caspase-1, IL-18 and IL-1β in the peri-ischemic region 3 days after MCAO. **(B–F)** Quantification results of NLRP3, ASC, CL-caspase-1, IL-18 and IL-1β protein levels in the ischemic cortex. Means ± SD. n = 5/group. ^a^*P* < 0.01 versus sham; ^b^*P* < 0.01 versus vehicle; ^c^*P* < 0.05 versus Rux.

To further determine the effect of Rux on neuroinflammation and neuronal death, we performed double immunostaining for GFAP (a marker of astrocytes), NeuN (a neuronal nucleus marker) or CD68 (a marker of activated macrophages and microglia) and NLRP3. The results illustrated that the NLRP3 inflammasome was activated in the ischemic penumbra in the cerebral ischemia-reperfusion group but not in the sham group (**Figures 2A, C** and **3A, C**). In addition, western blotting results indicated that Rux decreased the levels of NLRP3 inflammasome-related proteins, including NLRP3 (0.525 ± 0.06 vs 0.256 ± 0.07, *P* < 0.001), ASC (0.560 ± 0.07 vs 0.354 ± 0.07, *P* = 0.002), CL-caspase-1 (0.542 ± 0.09 vs 0.354 ± 0.03, *P* < 0.001), IL-β (0.476 ± 0.06 vs 0.284 ± 0.03, *P* < 0.001) and IL-18 (0.549 ± 0.06 vs 0.334 ± 0.06, *P* < 0.001), in cerebral ischemic tissues 3 days after MCAO compared with the vehicle group (**Figure 4**). We also counted NeuN^+^ cells and microglia/macrophages, as microglia/macrophages are pivotal indicators of neuroinflammation. The results indicated that there were few NeuN^+^ neurons (37.6 ± 5.13 vs 88.6 ± 4.04, *P* < 0.001) in the ischemic penumbra, whereas the number of activated microglia/macrophages (66.2 ± 4.21 vs 21.0 ± 3.01, *P* < 0.001) was increased 3 days after MCAO compared with the sham group (**Figures 2A, B** and **3A, B**). Immunofluorescence staining further demonstrated that the NLRP3 inflammasome was activated in both NeuN^+^ cells and microglia/macrophages 3 days after MCAO (**Figures 2D** and **3D**). Compared with vehicle administration, administration of Rux greatly limited the number of NLRP3^+^ cells (36.4 ± 3.21 vs 57.8 ± 6.06, *P* < 0.001) and microglia/macrophages (39.2 ± 5.54 vs 62.2 ± 4.21, *P* < 0.001) in the ischemic penumbra (**Figures 2A–C** and **3A, C**). These results indicated that Rux strongly suppressed neuroinflammation after IS. In addition, Rux treatment obviously alleviated neuronal death (66.2 ± 4.21 vs 37.6 ± 5.13, *P* < 0.001) (**Figures 3A, B**). MCC950 was used to verify the effect of Rux on the expression levels of NLRP3 inflammasome components. Similar to Rux, MCC950 markedly reduced the number of NLRP3^+^ cells (**Figures 2A, C** and **3A, C**) and suppressed the expression of NLRP3 inflammasome-related proteins, and the effect of MCC950 was not significantly different from that of Rux (**Figure 4**).

### Rux Treatment Suppresses the Infiltration of Macrophages, B, and T Cells

**Figure 5:**
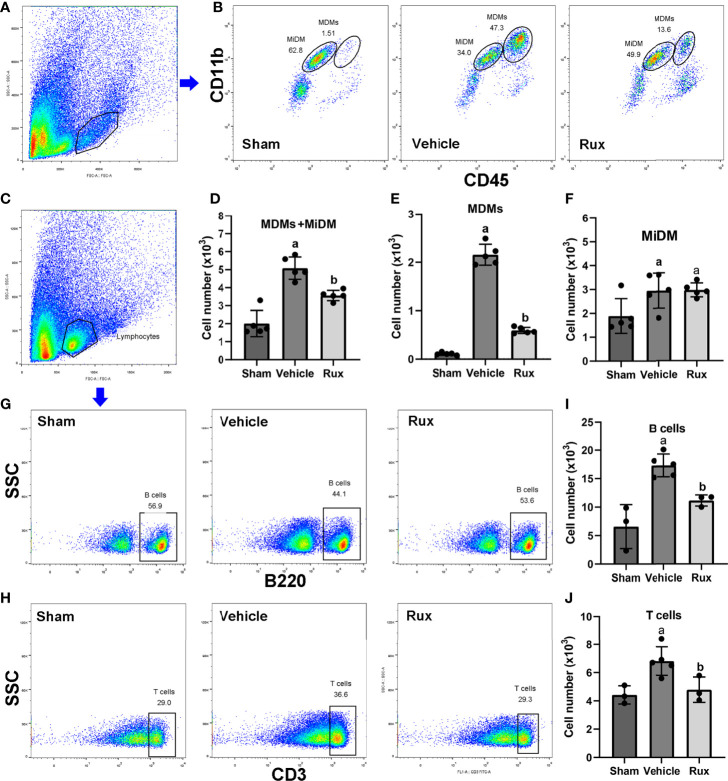
Rux administration reduced the infiltrated macrophages, B, and T cells. **(A, B)** Gating strategy to identify CD45^inter^CD11b^+^ (MiDM) and CD45^high^CD11b^+^ (MDMs) cells. **(D–F)** Bar graphs showing the results for CD45^inter^CD11b^+^ (MiDM) and CD45^high^CD11b^+^ (MDMs) cells. **(C, G, H)** Gating strategies for identifying B220^+^ B cells, and CD3^+^ T cells. **(I, J)** Bar graphs showing the results for B lymphocytes and T lymphocytes. n = 3 to 5 per group. ^a^*P* < 0.01 versus sham; ^b^*P* < 0.01 versus vehicle.

**Figure 6:**
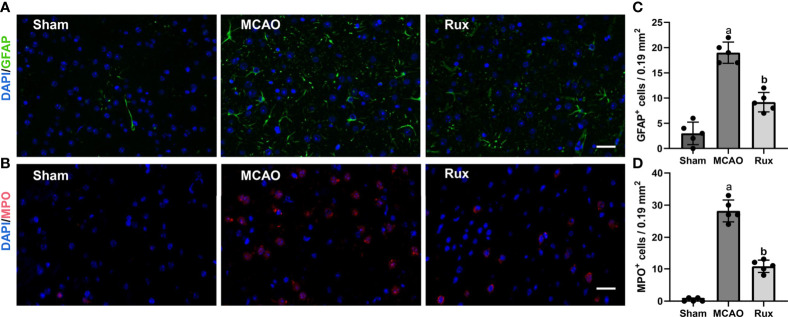
Rux treatment decreased the number of GFAP^+^ and MPO^+^ cells in the ischemic penumbra 3 days after MCAO. **(A, C)** Representative immunostaining of GFAP and MPO in the penumbra region. **(C, D)** Quantification results of GFAP^+^ and MPO^+^ cells in the peri-ischemic region. Means ± SD. n = 5. ^a^*P* < 0.01 versus sham; ^b^*P* < 0.01 versus vehicle; Scale bar: 50 µm.

As the CD68-positive cells in the brain are composed of monocyte-derived macrophages (MDMs) and microglia derived macrophages (MiDM). We used flow cytometry to further distinguish whether the CD68-positive cells were diminished due to reduced infiltration of peripheric macrophages or reduced central microglia. We found that Rux reduced the number of MDMs (CD45^high^CD11b^+^) cells compared with the vehicle group (0.593 ± 0.06 vs 2.156 ± 0.22, *P* < 0.001) (**Figures 5A, B, E**), indicating that Rux restrained the infiltration of peripheric macrophages. However, the number of MiDM was not reduced by Rux compared with the vehicle group (2.951 ± 0.13 vs 2.727 ± 0.64, *P* = 0.788) (**Figures 5B, F**). The total number of MDMs and MiDM was decreased by Rux administration compared with the vehicle group (3.544 ± 0.12 vs 4.866 ± 0.46, *P* = 0.002) (**Figures 5B, D**), which was consist with our results in **Figure 2**. In addition, Rux reduced the infiltration of B220^+^ B cells (11.14 ± 0.98 vs 17.30 ± 1.99, *P* = 0.02) (**Figures 5C, G, I**), and CD3^+^ T cells (4.80 ± 0.90 vs 6.83 ± 1.01, *P* = 0.037) (**Figures 5H, J**) compared with the vehicle. We also detected the astrocytes (GFAP^+^ cells) and neutrophils (MPO^+^ cells) by immunofluorescence in the ischemic penumbra, the results showed that the number of astrocytes (9.20 ± 1.92 vs 19.00 ± 2.12, *P* < 0.001) (**Figures 6A, C**) and infiltrated neutrophils (10.80 ± 1.92 vs 28.20 ± 3.41, *P* < 0.001) (**Figures 6B, D**) were also reduced compared with the vehicle group.

### Rux Reduces Proinflammatory Cytokine Expression After IS

**Figure 7:**
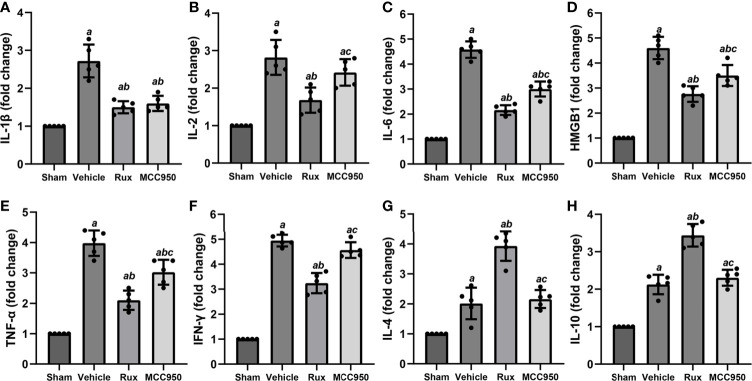
Rux treatment reduced the mRNA expression of proinflammatory cytokines 3 days after MCAO. **(A–F)** mRNA expression of IL-1β, IL-2, IL-6, HMGB1, TNF-α, and IFN-γ. **(G, H)** mRNA expression of IL-4 and IL-10. The data are shown as fold changes compared to the sham. n = 5. ^a^*P* < 0.001 versus sham; ^b^*P* < 0.01 versus vehicle; ^c^*P* < 0.05 versus Rux.

The levels of numerous proinflammatory cytokines are elevated by the JAK2/STAT3 pathway, and these cytokines play a crucial role in pathological injury in stroke. To determine whether Rux decreases proinflammatory cytokine expression by suppressing the JAK2/STAT3 pathway, the mRNA expression levels of proinflammatory cytokines (IL-1β, IL-2, IL-6, HMGB1, TNF-α, and IFN-γ) in the ischemic penumbra of the ischemic cortex were measured 3 days after MCAO. RT-qPCR analyses demonstrated that the mRNA levels of IL-1β (2.72 ± 0.43, *P* < 0.001), IL-2 (2.82 ± 0.47, *P* < 0.001), IL-6 (4.58 ± 0.33, *P* < 0.001), HMGB1 (4.60 ± 0.45, *P* < 0.001), TNF-α (3.98 ± 0.43, *P* < 0.001), and IFN-γ (4.95 ± 0.23, *P* < 0.001) increased in the peri-ischemic zone 3 days after MCAO compared with the sham group (**Figures 7A–F**). Compared with vehicle, Rux treatment suppressed the expression of IL-1β (1.5 ± 0.16 vs 2.72 ± 0.43, *P* < 0.001), IL-2 (1.68 ± 0.33 vs 2.82 ± 0.47, *P* < 0.001), IL-6 (2.16 ± 0.19 vs 4.58 ± 0.33, *P* < 0.001), HMGB1 (2.76 ± 0.32 vs 4.60 ± 0.45, *P* < 0.001), TNF-α (2.10 ± 0.32 vs 3.98 ± 0.43, *P* < 0.001), and IFN-γ (3.24 ± 0.41 vs 4.95 ± 0.23, *P* < 0.001) to a certain extent (**Figures 7A–F**). Furthermore, the mRNA levels of IL-2 (1.68 ± 0.33 vs 2.42 ± 0.36, *P* = 0.015), IL-6 (2.16 ± 0.19 vs 3.01 ± 0.30, *P* < 0.001), HMGB1 (2.76 ± 0.32 vs 3.50 ± 0.42, *P* = 0.017), TNF-α (2.10 ± 0.32 vs 9 vs 3.02 ± 0.41, *P* = 0.002), and IFN-γ (3.24 ± 0.41 vs 4.57 ± 0.31, *P* < 0.001) were more markedly suppressed in the Rux group than in the MCC950 group (**Figures 7B–F**). We also assessed the mRNA levels of anti-inflammatory cytokines (IL-4 and IL-10). The results showed that IL-4 (3.93 ± 0.49 vs 2.16 ± 0.30, *P* < 0.001) and IL-10 (3.44 ± 0.30 vs 2.31 ± 0.21, *P* < 0.001) expression was increased at the transcriptional level in the Rux-treated group compared with the vehicle-treated group (**Figures 7G, H**). These findings indicated that the administration of Rux not only suppressed NLRP3 inflammasome expression but also restrained the production of proinflammatory cytokines.

### Rux Downregulates MCAO-Induced P-JAK2/P-STAT3 Expression in Brain Tissues

**Figure 8:**
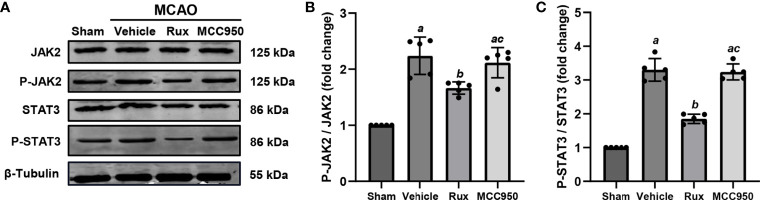
The administration of Rux inhibited the activation of P-JAK2 and P-STAT3 3 days after MCAO. **(A)** Representative western blots showing that Rux inhibited P-JAK2/P-STAT3 expression in the peri-ischemic region 3 days after MCAO. **(B, C)** Quantification of P-JAK2/P-STAT3 expression in the ischemic penumbra. Means ± SD, n = 5/group. ^a^*P* < 0.01 versus sham; ^b^*P* < 0.05 versus vehicle; ^c^*P* < 0.05 versus Rux.

As Rux is a JAK1/2 inhibitor, we hypothesized that the inhibitory effect of Rux on the activation of the NLRP3 inflammasome and the expression of proinflammatory cytokines may be achieved through the regulation of STAT3 activation. To explore the interplay between Rux, reductions in proinflammatory cytokine levels, and the NLRP3 inflammasome, we evaluated the expression of components of the JAK2/STAT3 pathway. As expected, western blot analysis illustrated that compared with vehicle, Rux treatment reduced P-JAK2 (1.66 ± 0.11 vs 2.24 ± 0.33, *P* = 0.004) and P-STAT3 (1.85 ± 0.14, 3.30 ± 0.33, *P* < 0.001) expression in the ischemic penumbra of the ischemic cortex 3 days after MCAO (**Figure 8**).

### P-STAT3 Deficiency Downregulates NLRP3 Expression by Inhibiting Histone H3 and H4 Acetylation of the NLRP3 Promoter After Stroke

**Figure 9:**
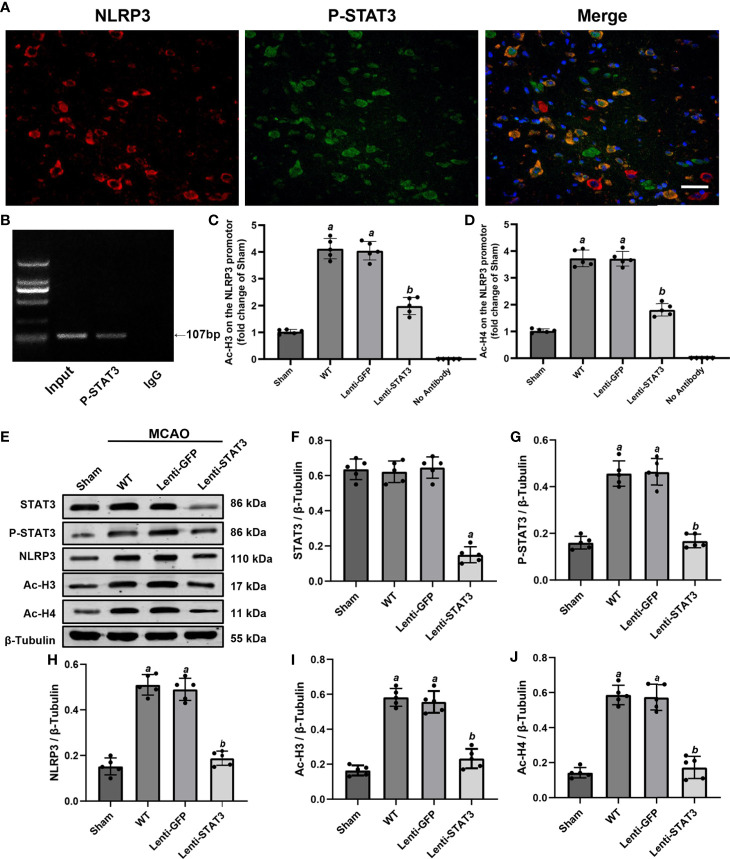
Local STAT3 deficiency decreased histone H3 and H4 acetylation and NLRP3 expression. **(A)** Representative immunostaining of NLRP3 and P-STAT3 in the penumbra of the ischemic cortex 3 days after MCAO. **(B)** ChIP assay indicating that P-STAT3 bound to the NLRP3 promotor. **(C, D)** ChIP-qPCR assays showing that Ac-H3 and Ac-H4 on the NLRP3 promotor were decreased by local STAT3 deficiency. **(E)** Representative western blots showing that STAT3 knockout restrained the expression of P-STAT3, NLRP3, and global Ac-H3 and Ac-H4 in the peri-ischemic region 3 days after MCAO. **(F–J)** Quantification of STAT3, P-STAT3, NLRP3, and global Ac-H3 and Ac-H4 levels in the ischemic penumbra. Means ± SD. n = 5/group. ^a^*P* < 0.001 versus sham; ^b^*P* < 0.05 versus lenti-GFP. Scale bar: 50 µm.

To further explore the direct connection between P-STAT3 and the NLRP3 inflammasome, we performed double immunostaining for P-STAT3 and the NLRP3 inflammasome. Immunofluorescence staining illustrated that NLRP3 was expressed in P-STAT3^+^ cells in the peri-ischemic region 3 days after MCAO (**Figure 9A**). ChIP assays also illustrated that P-STAT3 bound to the NLRP3 promoter 3 days after MCAO (**Figure 9B**). Western blot analysis showed that P-STAT3 and NLRP3 protein levels were elevated 3 days after IS in the WT (0.456 ± 0.05, *P* < 0.001;0.510 ± 0.05, *P* < 0.001) and lenti-GFP groups (0.464 ± 0.06, *P* < 0.001; 0.490 ± 0.05, *P* < 0.001) compared with the sham operation group (0.160 ± 0.03; 0.152 ± 0.04) (**Figures 9E, G, H**). Western blot analyses also demonstrated that local deficiency of STAT3 induced by lentivirus lenti-STAT3 (0.188 ± 0.03 vs 0.51 ± 0.05, *P* < 0.001) markedly suppressed the upregulation of NLRP3 protein expression in the WT group (**Figures 9E, F, H**).

A previous study illustrated that activation of STAT3 may upregulate NLRP3 expression by increasing the acetylation (Ac) of histone H3 and H4 on the NLRP3 promoter and the recruitment of STAT3 to the NLRP3 promoter in the dorsal root ganglion (36). To further verify whether P-STAT3 can directly regulate NLRP3 at the transcriptional level in the ischemic cortex after IS, we evaluated the expression of Ac-H3 and Ac-H4 on the NLRP3 promoter region in the ischemic cortex 3 days after IS. The western blot results showed that global ac-H3 (0.582 ± 0.05 vs 0.164 ± 0.03, *P* < 0.001) and ac-H4 (0.586 ± 0.06 vs 0.142 ± 0.3, *P* < 0.001) expression in the ischemic cortex was robustly elevated in the IS group compared with the sham group (**Figures 9E, I, J**). Additionally, compared with the lenti-GFP group, local knockdown of STAT3 by lenti-STAT3 decreased global ac-H3 (0.232 ± 0.06 vs 0.556 ± 0.06, *P* < 0.001) and ac-H4 (0.172 ± 0.06 vs 0.574 ± 0.07, *P* < 0.001) (**Figures 9E, I, J**). ChIP assays verified that STAT3 knockout in the brain suppressed the upregulation of H3 and H4 acetylation on the NLRP3 promotor (**Figures 9C, D**). These results demonstrated that P-STAT3 may regulate NLRP3 inflammasome expression by enhancing acetylation of histones H3 and H4 on the NLRP3 promoter.

### Rux Improves Cell Viability and Downregulates the Expression of NLRP3 Inflammasome-Related Molecules Through Inhibition of the JAK2/STAT3 Pathway After OGD/R *In Vitro*

**Figure 10:**
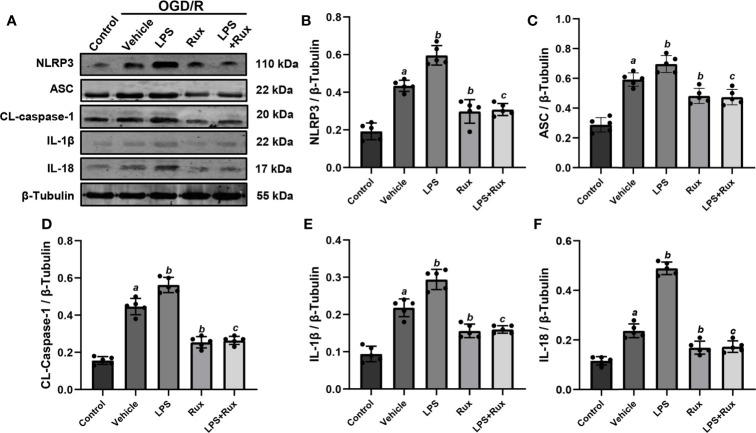
Administration of Rux reduced NLRP3 inflammasome activation in HT22 cells after OGD/R. **(A)** Representative western blots showing NLRP3, ASC, CL-caspase-1, IL-1β and IL-18 at the protein level. **(B–F)** Quantification results of NLRP3, ASC, CL-caspase-1, IL-1β and IL-18 expression at the protein level. Means ± SD. n = 5/group. ^a^*P* < 0.01 versus control; ^b^*P* < 0.05 versus OGD/R + vehicle; ^c^*P* < 0.05 versus OGD/R + LPS.

**Figure 11:**
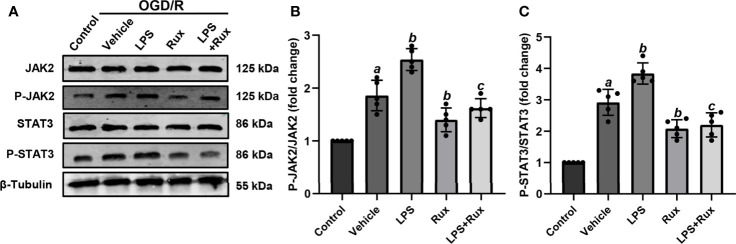
Rux treatment inhibited LPS-induced JAK2/STAT3 activation in HT22 cells after OGD/R. **(A)** Representative western blots showing that Rux administration downregulated P-JAK2/P-STAT3 expression in HT22 cells after OGD/R. **(B, C)** Quantification of P-JAK2 and P-STAT3 presented as fold changes. Means ± SD. n = 5/group. ^a^*P* < 0.01 versus control; ^b^*P* < 0.05 versus OGD/R + vehicle; ^c^*P* < 0.01 versus OGD/R + LPS.

Neuronal death can trigger the activation of microglia/macrophages (3). Therefore, we used HT-22 cells to establish an OGD/R model *in vitro* to further verify the protective action of Rux on CIRI. A CCK-8 assay was conducted to investigate the appropriate concentration of Rux for administration in HT-22 cells. The cell viability analyses showed that the IC50 of Rux in HT-22 cells was 4 µM (**Figure S3B**), which was chosen as the concentration for the subsequent experiments. Then, we established an OGD/R model in HT-22 cells *in vitro* and found that the optimal duration of OGD in HT-22 cells was 8 hours (**Figure S3A**). Finally, HT-22 cells were treated with the optimal concentration of Rux. We observed that the viability of HT-22 cells was markedly increased by Rux after OGD/R (**Figure S3C**). Moreover, western blotting analyses indicated that Rux significantly reduced the protein levels of NLRP3, ASC, CL-caspase-1, IL-1β and IL-18 in HT-22 cells in the OGD/R group compared with the vehicle + OGD/R group (**Figure 10**). LPS was applied to induce inflammation in HT-22 cells subjected to OGD/R, and we found that the administration of Rux markedly reduced the LPS-induced increase in NLRP3 inflammasome-related protein expression (**Figure 10**). LPS treatment increased P-JAK2 (2.54 ± 0.21 vs 1.86 ± 0.29, *P* < 0.001) and P-STAT3 (3.84 ± 0.37 vs 2.92 ± 0.41, *P* = 0.002) expression in HT22 cells after OGD/R compared with the vehicle group (**Figure 11**). However, compared with LPS alone, cotreatment with LPS and Rux restrained the expression of P-JAK2 (1.62 ± 0.18 vs 2.54 ± 0.21, *P* < 0.001) and P-STAT3 (2.20 ± 0.39 vs 3.84 ± 0.37, *P* < 0.001) (**Figure 11**). These results illustrated that Rux exerted a neuroprotective effect against CIRI by suppressing the NLRP3 inflammasome through inhibition of the JAK2/STAT3 pathway.

## Discussion

In this study, we found that JAK2 inhibition by Rux exerted a neuroprotective effect. The application of Rux markedly reduced the infarct size, neurological deficits and brain edema 3 days after MCAO. In addition, the expression of NLRP3 inflammasome-associated proteins and a number of proinflammatory cytokines in the penumbra of the ischemic cortex was inhibited by Rux 3 days after MCAO. Furthermore, we confirmed that CIRI promoted the phosphorylation of JAK2/STAT3, which was counteracted by Rux *in vivo* and *in vitro*. Local knockdown of STAT3 decreased ac-H3 and ac-H4 levels on the NLRP3 promoter and the expression of NLRP3. These findings indicated that JAK2 inhibition by Rux in MCAO mice decreased the phosphorylation of STAT3, thus inhibiting downstream proinflammatory cytokines and acetylation of H3 and H4 on the NLRP3 promoter, resulting in a limitation of NLRP3 inflammasome activation.

Rux, a selective oral JAK 1/2 inhibitor, is mainly used to treat polycythemia vera (6) and recalcitrant dermatomyositis (7), as well as myelofibrosis (8), due to its antitumor (9), immunosuppressive and anti-inflammatory functions (10). In addition, recent studies have illustrated that Rux has an anti-inflammatory effect in COVID-19 patients with severe systemic hyperinflammation governed by proinflammatory cytokines (5, 37). The key to the extensive anti-inflammatory activity of Rux is its ability to inhibit innate cytokine storm-associated downstream proinflammatory cytokines such as IL-1, IL-6, IL-8, TNF-α, and IFN-γ. The NLRP3 inflammasome is a trigger of the cytokine storm in severe infectious diseases such as SARS-CoV-2 infection (38) and COVID-19 (13). Proinflammatory cytokines exacerbate cerebral injury after IS. However, blood substitution therapy reduces the cytokine storm in the plasma and ameliorates IS injury in mice (39). Here, we revealed, for the first time, that administration of Rux suppresses the expression of NLRP3 inflammasome components and reduces numerous cytokine storm-associated proinflammatory cytokines, including IL-1β, IL-2, IL-6, IL-18, HMGB1, TNF-α, and IFN-γ, in MCAO mice, thereby exerting a neuroprotective effect after IS. These results suggest that the cytokine storm induced by proinflammatory cytokines is partially inhibited by NLRP3 inflammasome suppression resulting from the inhibition of the JAK2/STAT3 signaling pathway. In addition, the infiltrating B cells, T cells, MDMs, and neutrophiles were restrained by Rux, which partly explained the decrease of pro-inflammatory cytokines. As the pro-inflammatory cytokines are often produced by multiple cells, such as neurons, microphages, astrocytes, and lymphocytes (40, 41). We confirmed that the NLRP3 inflammasome was present in both neuronal and activated macrophages and microglial cells in the ischemic cortex, which is consistent with our previous study (15). These findings presumably suggest that the cytokine storm induced by proinflammatory cytokines, such as IL-1, IL-6, IL-8, IL-12, TNF-α and IFN-γ, is activated *via* NLRP3 inflammasome and JAK2/STAT3 signaling activation.

Inflammatory reactions in the peri-ischemic region are associated with the JAK2/STAT3 pathway, which in turn exacerbates cerebral injury. Additionally, our previous study showed that suppression of the NLRP3 inflammasome protects the brains of mice from CIRI (15). However, at present, whether the NLRP3 inflammasome can be regulated directly *via* the JAK2/STAT3 pathway after IS remains unclear. This study demonstrated that upregulation of JAK2/STAT3 expression was relieved by Rux in MCAO mice and LPS-treated HT22 cells after OGD/R. Furthermore, local STAT3 deficiency robustly blocked the upregulation of NLRP3 protein expression. P-STAT3 is known to translocate to the nucleus to directly regulate the transcription of target genes by binding to DNA. A previous study confirmed that STAT3 enhances histone acetylation by recruiting histone acetyltransferase to gene promoters, hence promoting transcription (42). Moreover, histone acetylation is widely recognized to regulate proinflammatory cytokines such as IL-8 (43). In this work, we assessed whether the NLRP3 inflammasome was directly regulated by P-STAT3 in the ischemic penumbra of MCAO mouse brains. The results revealed that in the brains of MCAO mice, downregulation of P-STAT3 expression by injection of a lentivirus reduced ac-H3 and ac-H4 levels on the NLRP3 promoter, which was consistent with previous research showing that activation of STAT3 elevates histone H3 and H4 acetylation on the NLRP3 promoter in dorsal root ganglion (36). These findings illustrate for the first time the regulatory effect of STAT3 signaling on the NLRP3 inflammasome in the MCAO mouse model.

However, the role of STAT3 signaling in inflammatory reactions remains controversial because it has been reported that activation of STAT3 contributes to the anti-inflammatory effects of some anti-inflammatory agents (44, 45). Therefore, the definite effect of STAT3 on CIRI needs to be further studied. A limitation of this work is that we mainly focused on the anti-inflammatory effect of Rux rather than its other roles, such as its angiogenic effect after IS through which it rescues cerebral ischemia injury; this could be investigated by measuring the expression of other cytokines downstream of JAK2/STAT3, G-CFS cytokines, growth factors and VEGF (46). In that case, the efficacy of Rux in the MCAO mouse model might be attributed to its pleiotropic actions. In addition, prophylactic administration of Rux also exerted a therapeutic effect similar to therapeutic administration. However, we have not explored the specific mechanisms involved. It is likely that prophylactic administration of Rux suppresses the peripheral inflammatory response and immune cells such as macrophages and lymphocytes similar to therapeutic administration, leading to a decrease in inflammatory cytokines and immune cells infiltrating the brain after MCAO. Hence, the neuroprotective effects, as well as other potential effects including on angiogenesis, of Rux in IS require further clarification.

## Conclusion

Taken together, our results indicate that Rux exhibits a neuroprotective effect against CIRI by relieving neuroinflammation *via* inhibition of the NLRP3 inflammasome through inactivation of the JAK2/STAT3 pathway. In addition, the NLRP3 inflammasome may be directly regulated by P-STAT3-dependent histone acetylation. Furthermore, NLRP3 inflammasome activation after stroke may be related to the induction of cytokine storm-related proinflammatory cytokines, which can be relieved by Rux *via* its targeting of the JAK2/STAT3 pathway. Hence, Rux may be a potential agent for IS treatment.

## Data Availability Statement

The original contributions presented in the study are included in the article/**Supplementary Material**. Further inquiries can be directed to the corresponding authors.

## Ethics Statement

The animal study was reviewed and approved by the Ethics Committee of Renmin Hospital of Wuhan University.

## Author Contributions

XX and LG designed the experiments. All the authors contributed to the performance of the experiments. ZJ, YY, YGZ, and BP analyzed the data. HZ, YZ, and XH wrote the article. All authors contributed to the article and approved the submitted version.

## Funding

This work was supported by the National Natural Science Foundation of China (no. 81870939 to Xiaoxing Xiong, no. 81771283 and 82071339 to LG) and the Natural Science Foundation of Hubei Province, China (no. 2019CFB498 to LG).

## Conflict of Interest

The authors declare that the research was conducted in the absence of any commercial or financial relationships that could be construed as a potential conflict of interest.

## Supplementary Material

Supplementary Figure 1 The death and survival rates of mice in the sham-, vehicle- and Rux-treated groups within 3 days after surgery. **(A)** The mortality rate of sham-, vehicle- and Rux-treated mice. **(B)** The survival rate of sham-, vehicle- and Rux-treated mice. Means ± SD. n = 10. ^a^*P* < 0.05 versus the sham group; ^b^*P* < 0.05 versus the vehicle group; ^c^*P* < 0.05 versus the 60 mg/kg Rux group.

Supplementary Figure 2 Both prophylactic and therapeutic administration of Rux (90 mg/kg) reduced infract volume. **(A)** Representative TTC-stained slices showing infarction in vehicle- and Rux-treated mice. **(B)** Quantitative analysis of the infarct size in prophylactic and therapeutic treatment group is presented as a percentage of the contralateral hemisphere size 3 days after stroke. **(C)** Neurological scores were determined 3 days after MCAO. ns, no significant difference.

Supplementary Figure 3 Rux increased the viability of HT-22 cells after OGD/R *in vitro*. **(A)** The appropriate duration of OGD in HT-22 cells was 8 hours. **(B)** The IC50 of Rux in HT-22 cells was 4 μM. **(C)** The viability of HT-22 cells treated with different concentrations of Rux after OGD/R. Means ± SD. n = 5. **P* < 0.05 versus the 0 h OGD group; ^#^*P* < 0.01 versus the 0 μM Rux group. ^a^*P* < 0.05 versus the OGD group.

## Abbreviations

Rux, Ruxolitinib; IS, Ischemic stroke; NLRP3, Nucleotide-binding oligomerization domain-like receptor protein 3; JAK2, Janus kinase 2; STAT3, Signal transducer and activator of transcription 3; P-STAT3, phosphorylated STAT3; MCAO, Middle cerebral artery occlusion; NOX4, NADPH oxidase 4; TNF-α, Tumor necrosis factor-α; IFN-γ, Interferon-γ; HMGB1, High mobility group box-1; IL, Interleukin; GM-CSF, Granulocyte-macrophage colony-stimulating factor; COVID-19, Coronavirus disease 2019; OGD/R, Oxygen-glucose deprivation/reperfusion; ASC, Apoptosis-associated speck-like protein containing a CARD; LPS, lipopolysaccharide; FBS, Fetal bovine serum.
