# Supplementary figures and images for: Janus Kinase Inhibition Ameliorates Ischemic Stroke Injury and Neuroinflammation Through Reducing NLRP3 Inflammasome Activation via JAK2/STAT3 Pathway Inhibition
Source: Front Immunol. 2021 Jul 22;12:714943. doi: 10.3389/fimmu.2021.714943 (PMC8339584; doi:10.3389/fimmu.2021.714943)

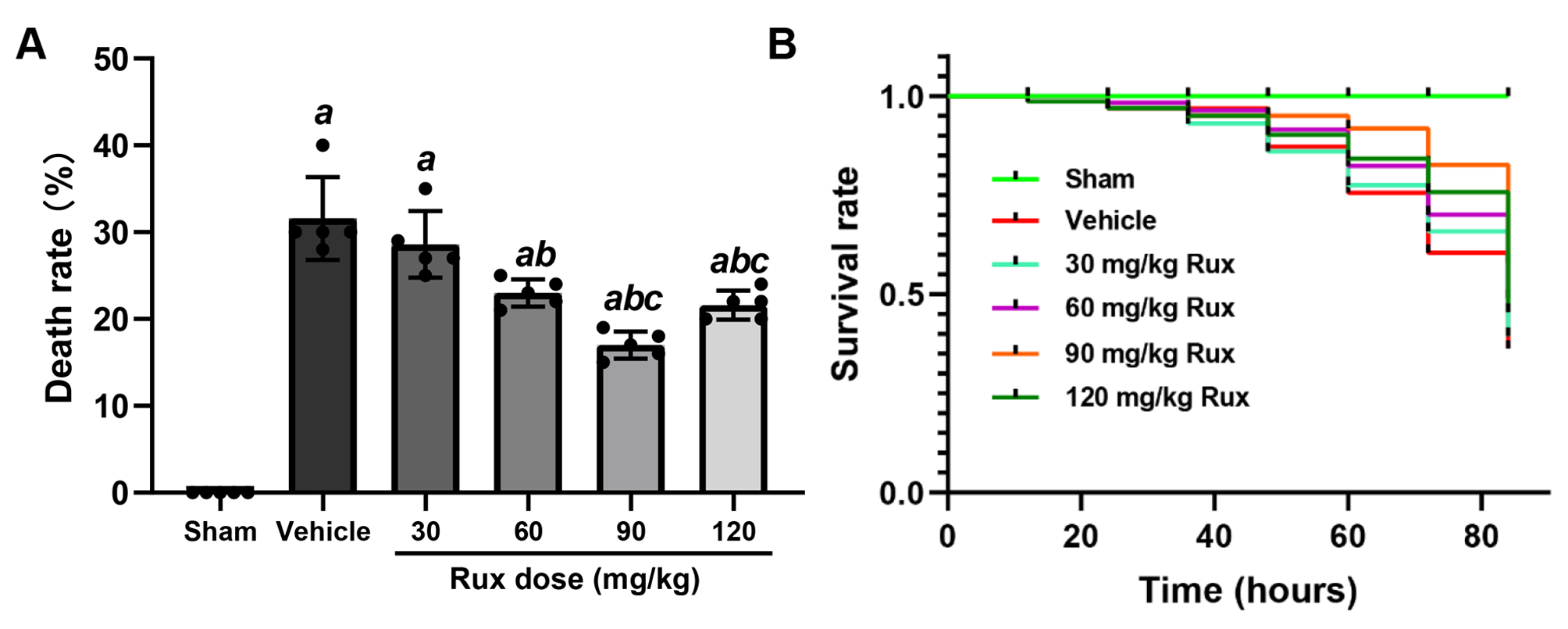

Supplement: Supplementary Figure 1 — The death and survival rates of mice in the sham-, vehicle- and Rux-treated groups within 3 days after surgery. (A) The mortality rate of sham-, vehicle- and Rux-treated mice. (B) The survival rate of sham-, vehicle- and Rux-treated mice. Means ± SD. n = 10. aP < 0.05 versus the sham group; bP < 0.05 versus the vehicle group; cP < 0.05 versus the 60 mg/kg Rux group. [file Image_1.tif]

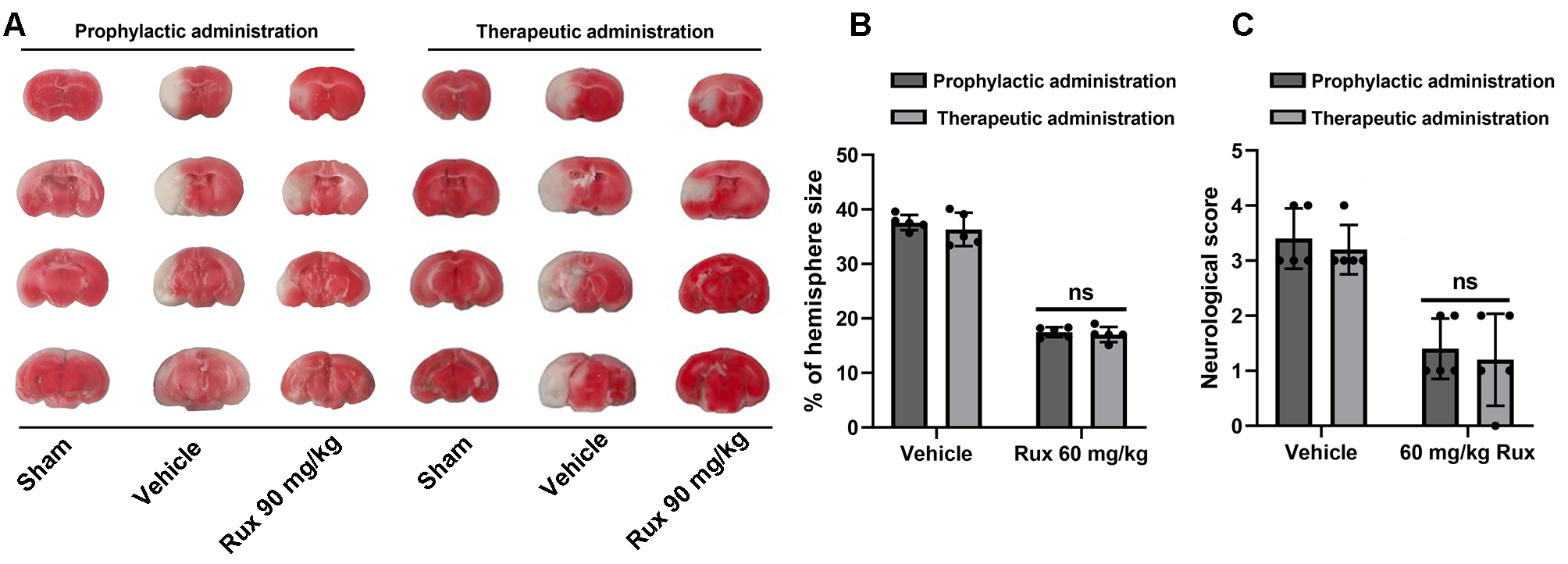

Supplement: Supplementary Figure 2 — Both prophylactic and therapeutic administration of Rux (90 mg/kg) reduced infract volume. (A) Representative TTC-stained slices showing infarction in vehicle- and Rux-treated mice. (B) Quantitative analysis of the infarct size in prophylactic and therapeutic treatment group is presented as a percentage of the contralateral hemisphere size 3 days after stroke. (C) Neurological scores were determined 3 days after MCAO. ns, no significant difference. [file Image_2.tif]

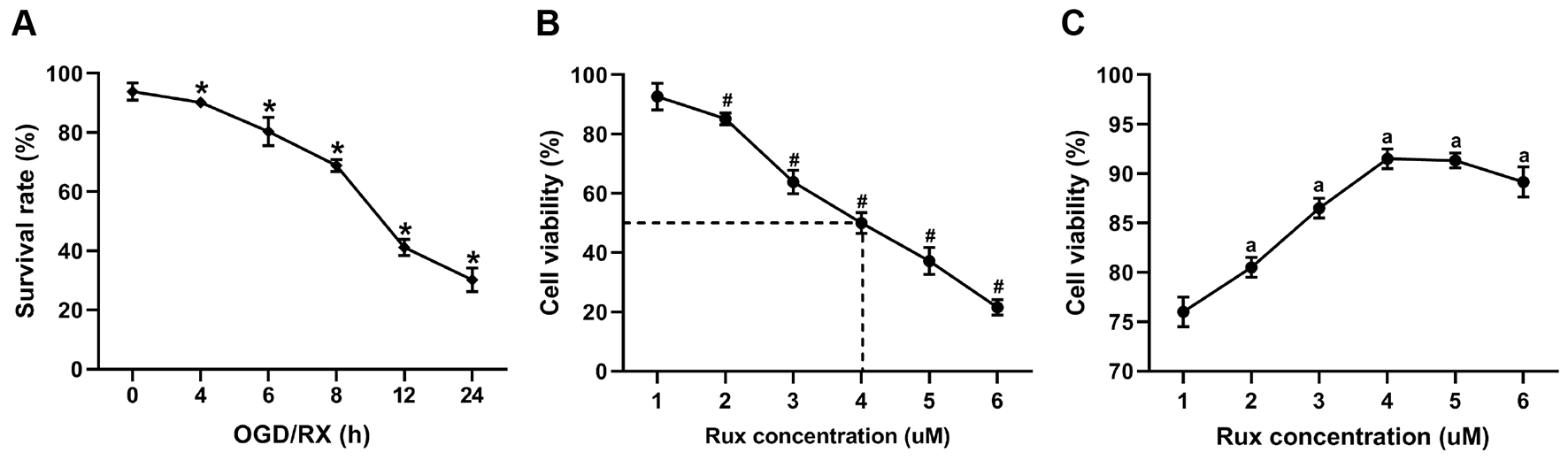

Supplement: Supplementary Figure 3 — Rux increased the viability of HT-22 cells after OGD/R in vitro. (A) The appropriate duration of OGD in HT-22 cells was 8 hours. (B) The IC50 of Rux in HT-22 cells was 4 μM. (C) The viability of HT-22 cells treated with different concentrations of Rux after OGD/R. Means ± SD. n = 5. *P < 0.05 versus the 0 h OGD group; #P < 0.01 versus the 0 μM Rux group. aP < 0.05 versus the OGD group. [file Image_3.tif]
